# The Impact of Radiotherapy on the Uterus and Its Implications for Pregnancy

**DOI:** 10.1055/s-0045-1809040

**Published:** 2025-05-14

**Authors:** Anne-Lotte L.F. van der Kooi, Wendy van Dorp

**Affiliations:** 1 Division of Reproductive Endocrinology and Infertility, Department of Obstetrics and Gynecology, Rotterdam, The Netherlands

**Keywords:** cancer, radiotherapy, uterus, pregnancy, survivorship

## Abstract

Radiotherapy is an effective treatment for various cancers, but it can cause significant side effects on various organ systems, including the reproductive organs, which is a major concern for women of reproductive age. A well-known long-term effect of oncological treatment is premature ovarian insufficiency. Another critical but sometimes overlooked organ at risk in female cancer survivors is the uterus. This review focuses on the impact of radiotherapy on uterine physiology, highlighting key issues such as the development of fibrosis and loss of elasticity, vascular damage, and hormonal disruption, all of which can compromise uterine function. These changes can negatively impact fertility and pregnancy outcomes, such as miscarriage, preterm birth, and low birth weight. Limited evidence is also available suggesting that radiotherapy may affect endometrial receptivity and contribute to abnormal placentation. We conclude by discussing strategies aimed at mitigating the damage caused by radiotherapy, such as fertility-preserving treatments and hormonal interventions. A thorough understanding of these effects is essential for healthcare providers to offer informed support to women who wish to maintain their fertility and have children following cancer treatment.

Radiotherapy is a well-established treatment modality for various cancers, including those affecting the pelvic region, such as cervical, ovarian, and endometrial cancers. While effective in targeting malignant cells, radiation therapy often carries significant side effects, particularly when the treatment field involves the reproductive organs. 1 One of the major concerns for women undergoing pelvic radiotherapy is the effect of radiation on fertility and pregnancy outcomes. The reproductive organs, being particularly radiosensitive, are susceptible to radiation-induced damage, which can result in long-term consequences for reproductive health.

A well-known long-term effect of oncological treatment is premature ovarian insufficiency (POI). 2 Another critical but sometimes overlooked organ at risk in female cancer survivors is the uterus, particularly when the patient is of reproductive age and desires future pregnancies. This review aims to explore the impact of radiotherapy on the uterus, examining its physiological and structural effects, the potential consequences for fertility and pregnancy, and the available strategies for mitigating these effects. By understanding the specific mechanisms of radiation damage, healthcare providers can better support women who wish to maintain their fertility and pursue pregnancy after cancer treatment.

## Radiotherapy and Uterine Physiology

Radiotherapy works by delivering high doses of ionizing radiation to kill cancerous cells, but this can also affect healthy surrounding tissues. The uterus, when exposed to radiation during the treatment of pelvic cancers, undergoes significant changes that can impair its normal physiological function. 3 These changes can be divided into three main categories: fibrosis, vascular damage, and hormonal disruption.

### Fibrosis and Loss of Elasticity

One of the most significant consequences of radiotherapy is the induction of fibrosis in the uterine tissues. Fibrosis refers to the excessive formation of fibrous connective tissue, resulting from the inflammatory response initiated by radiation exposure. The radiation causes damage to the cellular DNA and disrupts normal tissue repair mechanisms, leading to the deposition of collagen and other extracellular matrix proteins. 3 This leads to a loss of elasticity in the uterine walls, which may impair their ability to stretch and accommodate a growing fetus during pregnancy.

Already in 1999, uterine volume was shown to be decreased in 12 pediatric cancer survivors treated with total body irradiation (TBI), prior to which they had received chemotherapy. At adulthood, these women all had below-average small uteri, with a median uterine volume of −2.6 standard deviation scores (range: −6.3 to −0.6), despite the fact that 8 of the 12 had received HRT. 4 In a more recent study in Dutch adult childhood cancer survivors (CCSs), median uterine volume was 41.4 (interquartile range: 18.6–52.8) mL for 55 RT-exposed CCS and 61.3 (49.1–75.5) mL for 110 general population controls. 5 An Italian article reported reduced ovarian volumes after cancer treatment in comparison with controls. Patients treated with chemotherapy-only had a larger uterine volume (percentage volume reduction: 24.3%; 95% confidence interval [CI]: 3.2–41.9) than those treated with bone marrow transplant with or without concomitant chemotherapy or radiotherapy (percentage volume reduction: 71.1%; 95% CI: 64.9–76.6; *p* = 0.003). Patients undergoing hormone therapy, an indicator of POI, had smaller uterine sizes compared to those not receiving sex hormones. 6 In addition, age at primary treatment showed a positive correlation with uterine volume, showing that especially younger pediatric patients were at risk of smaller uterine volumes, as had also been described previously. 7 The uterine developmental stage in premenarcheal girls appears to be particularly vulnerable to radiation-induced damage, a problem that sex hormones could partially remedy, 7 but only to a limited extent.

The level of fibrosis correlates with the radiation dose, with higher doses resulting in more pronounced fibrotic changes. 7 For example, doses above 45 Gy (Gray units) are often associated with irreversible uterine damage, although exceptions have been reported. 8 Uterine fibrosis can cause complications such as uterine rupture during pregnancy, increased risk of miscarriage, and preterm labor due to the reduced capacity of the uterus to expand as the pregnancy progresses. 4

### Vascular Damage and Ischemia

Radiotherapy can also lead to damage in the blood vessels supplying the uterus, including the endometrium and myometrium. Healthy vasculature is critical for maintaining an appropriate uterine environment, particularly during pregnancy, when the demand for blood flow increases significantly to support placental function and fetal growth. Radiation can cause endothelial damage, leading to the thickening of blood vessel walls and reduced blood flow to the uterine tissues. 9

Radiotherapy-induced vascular damage can lead to uterine ischemia, which in turn reduces the availability of oxygen and nutrients to the endometrium and myometrium. This ischemic environment may impair the ability of the uterus to support implantation, leading to infertility, recurrent miscarriage, and fetal growth restriction (FGR) in pregnancies that do progress beyond the first trimester.

For instance, in the article by Holm et al., 4 uterine blood flow was impaired, as a systolic blood flow could be measured in six of nine individuals, and a diastolic blood flow in one of nine females. The hormone replacement therapy that these young women had received was sufficient to induce bleeding and suppress other stigmata of POI but was inadequate to generate normal uterine growth and blood flow. The study by Beneventi et al. demonstrated that, in comparison to healthy controls, the pulsatility index of the uterine artery was significantly higher in individuals who received TBI, with a percentage increase of 35.6% (95% CI: 21.9–51.6; *p* < 0.001). 6

### Hormonal Disruption

Even if the primary focus of radiotherapy is the direct effect on uterine tissue, it can also disrupt the hormonal environment necessary for maintaining uterine function. More often, radiotherapy will be focused on a specific abdominal organ, and scatter radiation will possibly affect both the uterus and the ovaries. In the case of TBI, both reproductive organs are affected as well. Radiotherapy to areas exposing the ovaries or the hypothalamic–pituitary–ovarian axis can lead to low estrogen and progesterone levels, hormones critical for preparing the endometrium for implantation and maintaining pregnancy. In women who retain some ovarian function postradiotherapy, hormonal insufficiency may still occur, leading to an atrophic endometrium. A thin, underdeveloped endometrial lining is less capable of supporting embryo implantation, which further exacerbates infertility and miscarriage risk. Importantly, hormonal imbalances can contribute to irregular menstrual cycles and anovulation, which can be a sign of POI. As this can go hand in hand with estrogen deprivation, sex hormone replacement therapy should be considered. 2 10

### Childhood Cancer Survivors

In summary, radiotherapy during childhood, particularly before menarche, significantly increases the risk of impaired uterine function, as outlined earlier. The uterine developmental stage in premenarchal girls seems especially susceptible to radiation-induced damage. While sex hormones may offer some degree of remedy, 7 their effectiveness remains limited.

## Fertility Outcomes after Radiotherapy

Women of reproductive age undergoing pelvic radiotherapy face significant challenges in preserving fertility and achieving successful pregnancies. The effects of radiotherapy on reproductive potential are multifaceted, involving both direct damage to the uterus and indirect effects on the ovaries and hormonal regulation. The toxicity of radiotherapy on the ovaries and its hormonal effects are not explored in depth, as they fall outside the primary scope of this article, which centers on the uterus. However, in the context of fertility, it is important to address a few key points.

Infertility is one of the most significant long-term effects of pelvic radiotherapy. Women who were treated with radiotherapy to which the ovaries were potentially exposed are at an increased risk for developing POI. 11 12 13 The ovarian reserve, which refers to the pool of remaining viable oocytes in a woman's ovaries, is highly susceptible to radiation. The risk of infertility also depends on the woman's age at the time of treatment. Younger women generally have a higher ovarian reserve and may be more likely to retain some reproductive function postradiotherapy. Women over 35 years of age are at a higher risk of ovarian insufficiency following radiation therapy, as their baseline ovarian reserve is already diminished. The risk of infertility also increases with higher radiation doses and broader treatment fields that encompass both the uterus and the ovaries. A clear threshold for a safe radiotherapy dose has not been defined. 2 Mathematic modeling based on oocyte decline data suggests that the effective sterilizing dose is 20.3 Gy in infants, 18.4 Gy at the age of 10 years, and 16.5 Gy at the age of 20 years. 14 15 Abdominal radiation dosimetry and individual chemotherapy predictors are thus important predictors in age-specific models for developing POI. 16

## Pregnancy Outcomes after Radiotherapy

**Fig. 1:**
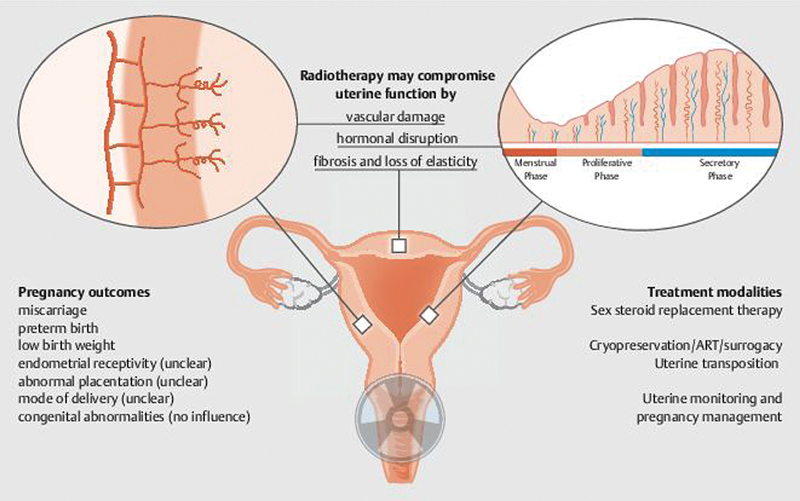
Summary of effects of radiotherapy on the uterus.

Women who conceive after undergoing pelvic radiotherapy are at an increased risk of pregnancy complications, even if fertility is preserved. These complications arise from radiation-induced damage to the uterine structure and vasculature, which could impair the uterus's ability to support a pregnancy ( Fig 1 ).

### Miscarriage

Miscarriage rates are significantly higher in women who have undergone pelvic radiotherapy. The combination of endometrial damage, uterine fibrosis, and poor vascularization makes it difficult for embryos to implant successfully and for pregnancies to progress beyond the first trimester.

The definition of miscarriage varied among reported studies, typically encompassing pregnancies that ended before 20 weeks of gestation or, in the context of the British Childhood Cancer Survivor Study, before 24 weeks. Three large cohorts of survivors of pediatric cancer reported increased risks with regard to miscarriage. Relative risks on self-reported miscarriages of 1.73 (1.49–2.00) compared to siblings were reported in the North American Childhood Cancer Survivor Study, 17 with similar findings in a study from Australia (which documented registered threatened miscarriage occurring after 20 weeks), 18 and a Danish study (which recorded registered spontaneous abortion without further specification). 19 Although there is evidence suggesting a dose–response relationship between radiotherapy exposure and risks associated with uterine exposure, a safe threshold dose remains undetermined. While no specific measures have been established to mitigate this risk, survivors should be counseled regarding their potentially heightened risk of miscarriage. 20

### Preterm Labor and Birth

The loss of elasticity in the uterine wall due to fibrosis can lead to preterm labor. Preterm labor occurs when the uterus begins contracting before the fetus has reached full term, often resulting in the delivery of a premature infant. Preterm birth is associated with various neonatal complications, including respiratory distress, developmental delays, and increased rates of infant mortality. Research shows that women exposed to pelvic radiotherapy are over two times more likely to experience preterm birth compared to the general population. 21 22 23 24 It should be noted, however, that another study including 582 women treated with radiotherapy did not find an increased risk 25 and one found an increased risk in those treated with radiotherapy only, but not in survivors treated with radiotherapy in combination with chemotherapy. 18

In addition to the loss of uterine elasticity, preterm labor can also occur due to iatrogenic factors. A large retrospective cohort study conducted in the United States, which included commercially insured cancer survivors, found that the incidence of preterm labor was 14.8% among cancer survivors compared to 12.4% among women without a cancer history (adjusted risk ratio: 1.19, 95% CI: 1.06–1.34). Not surprisingly, cancer survivors exhibited a higher prevalence of maternal comorbidities both prior to and during pregnancy. Approximately 26% of the association between cancer and preterm birth was mediated by these comorbidities. 26

### Low Birth Weight and Fetal Growth Restriction

Uterine ischemia resulting from vascular damage may restrict blood flow to the fetus, leading to FGR. Babies born with FGR are more likely to have low birth weights and experience complications such as hypoglycemia, difficulty breathing, and long-term developmental issues. An increased risk of FGR after radiotherapy was not detected in one study, 27 but high-dose radiation exposure to the uterus was found to be statistically significantly associated with an elevated risk of low birth weight in another. 23 Among the offspring of survivors who did not undergo any radiotherapy, only 7.6% had a birth weight <2,500 g and were categorized as low birth weight. In stark contrast, 25.5% of the children of survivors who received uterine radiation doses between 250 and 500 cGy were born with a low birth weight (odds ratio [OR] = 4.3, 95% CI = 1.4 to 12.8; *p* = 0.01). Furthermore, a substantial 36.2% of the children of survivors exposed to uterine doses exceeding 500 cGy were similarly classified as low birth weight (odds ratio = 6.8, 95% CI= 2.1 to 22.2; *p* = 0.001). 23

Other studies showed relative risks on low birth weight ranging from 1.38 (95% CI: 1.03–1.85) after any radiotherapy versus controls 28 to 2.31 (95% CI: 1.50–3.55) after abdominal radiotherapy in comparison to survivors not treated with radiotherapy, 29 to even increased odds ratio of 15.7 (95% CI: 1.43–171.35) in a cohort of 55 radiated survivors compared to 110 population controls. 5 These odds are high, but the large CI must be noted.

All in all, the heightened likelihood of low birth weight and FGR is not merely theoretical; it has been documented in diverse populations across multiple studies. This evidence underscores the real-world implications of these conditions, emphasizing the importance of monitoring and addressing these risks in affected groups.

### Endometrial Receptivity, Decidualization, and Placental Abnormalities

Placental abnormalities, such as placenta previa (where the placenta implants in the lower part of the uterus) and placental abruption (premature detachment of the placenta), are more common in women who have undergone pelvic radiotherapy. These conditions can possibly lead to life-threatening hemorrhage during pregnancy and delivery, placing both the mother and fetus at significant risk.

Griffiths et al showed the importance of radiotherapy on the uterus in mouse models. They transferred donor mice's embryos into ovariectomized mice that had been exposed to TBI, and compared their outcome with mice not treated with radiotherapy. Blastocyst attachment, implantation, and cytokines were similar between both groups, but blood-vessel area was significantly reduced in the irradiated mice. The uteri of radiated mice also showed a reduced decidualization response. Decidualization involves the differentiation of endometrial stromal cells into large, round decidual cells. It is hormone-driven and essential for ongoing pregnancy and early placental development. In the radiated mice, transcript levels of the hormonally regulated target gene and key regulator of decidualization *Bmp2* were significantly reduced. This mouse model showed that TBI impaired the decidual response in mice, which can lead to failed placental development. TBI also caused uterine artery endothelial dysfunction, likely preventing adequate blood vessel remodeling in early pregnancy. 30

In a comparative study of oocyte donation outcomes, 142 women previously treated and cured of cancer underwent 333 cycles of oocyte donation, while the control group consisted of 17,844 women who experienced 29,778 cycles. It must be noted that like most retrospective cohort studies, this population consisted of a mix of treatments with often chemotherapy including radiotherapy and also partially chemotherapy without radiotherapy. Subanalysis often yields power problems in these studies. In this Spanish study, the cancer survivors received a higher average number of donated oocytes (11.5) compared to the controls (10.9) ( *p* < 0.05), although the average number of embryos transferred was lower in the cancer group (1.89 vs. 2.08; *p* < 0.05). While the pregnancy rate in the first cycle was also significantly reduced for the cancer survivors (48.2 vs. 57.7%; *p* = 0.029), key parameters such as implantation rates, total deliveries, and cumulative pregnancy and delivery rates across all cycles did not exhibit significant differences, suggesting that endometrial receptivity may be comparable between the two groups with and without treatment for cancer. 31

Placental abnormalities are rarely reported in epidemiological studies. A relatively small Dutch study had found an increased risk in abdominally radiated survivors on postpartum hemorrhage. 32 However, this finding was not corroborated in a large Finnish population study. 25 32 Another analysis from the British Childhood Cancer Survivor Study also reported no increased risk after adjustment for confounding (RR: 1.33; 95% CI: 0.84–1.07) compared to survivors not treated with any radiotherapy. 29

### Mode of Delivery

Studies that investigated the risk of emergency cesarean sections following radiotherapy or abdominal radiotherapy reported no significant increase in risk. 25 29 This may indicate that, at least within the populations studied, prior exposure to radiotherapy does not appear to adversely affect the likelihood of requiring an emergency cesarean delivery. This lack of association is particularly noteworthy, as it alleviates concerns about the potential complications that may arise from previous radiotherapy treatment in pregnant individuals. Understanding the implications of such treatments on obstetric outcomes is crucial for healthcare providers, as it informs counseling and management strategies for cancer survivors during their pregnancies.

Overall, these studies contribute to a growing body of literature suggesting that while radiotherapy can have various effects on reproductive health, the specific risk of emergency cesarean sections does not seem to be one of them. Further research may continue to explore this relationship, ensuring that pregnant individuals with a history of radiotherapy receive appropriate care and support throughout their pregnancies.

### Congenital Abnormalities

While many have feared the possible mutagenic effects on germ cells, research clearly indicates that radiotherapy imposes no increased risk of congenital abnormalities. A meta-analysis of five studies that had reported incidence numbers of congenital abnormalities after high-risk radiation showed a nonsignificant relative risk of 1.18 (95% CI: 0.77–1.79) 24 in keeping with the statistically nonsignificant reported risks or ORs in all the source articles. 32 33 34

## Preserving Fertility and Pregnancy Outcomes

Given the significant risks associated with pelvic radiotherapy, several strategies have been developed to help women preserve their fertility and improve pregnancy outcomes. 35 These strategies focus on protecting ovarian and uterine function during treatment, as well as offering reproductive assistance posttreatment.

Fertility preservation is a critical consideration for women of reproductive age who are about to undergo cancer treatment. Several methods can be employed to protect the ovaries and reduce the impact of radiation on reproductive potential.

### Cryopreservation

Cryopreservation of oocytes, embryos, or ovarian tissue is the standard care for women who wish to preserve their fertility. This technique allows women to undergo assisted reproductive technologies (ARTs), such as in vitro fertilization (IVF), after cancer treatment. While cryopreservation does not protect the uterus from radiation damage, it provides an opportunity for conception if ovarian function is compromised. For prepubertal girls at high risk for POI, cryopreservation of ovarian tissue is the only option as the dormancy of their hypothalamic–pituitary axis still lies dormant, inhibiting the possibility of cryopreservation of oocytes, let alone embryos. 36 37 Cryopreservation of ovarian tissue can be obtained by either a unilateral ovariectomy or biopsy. 38 39

### Assisted Reproductive Technologies

Women who experience infertility or difficulty conceiving postradiotherapy may benefit from ART. IVF is one of the most commonly used methods to assist women in achieving pregnancy. IVF bypasses some of the barriers to natural conception by fertilizing an oocyte outside the body and then implanting the embryo into the uterus. For women with significant uterine damage, surrogacy may also be an option, where a surrogate carries the pregnancy to term using the couple's fertilized embryo. While surrogacy can overcome the limitations of a damaged uterus, it may not be a feasible or accessible option for all patients due to legal, ethical, and possibly financial considerations.

### Uterine Transposition

A more experimental approach is the option of uterine transposition. In a small study of eight patients, uterine transposition at a median age of 30.5 years (range: 19–37) was performed. The uterus was successfully preserved in six patients, accompanied by normal menses, hormonal levels, and vaginal intercourse after treatment. Cervical ischemia was the most common postsurgical complication in three (37.5%) patients. Three patients attempted to conceive, and two (66%) were spontaneously successful and delivered healthy babies at 36 and 38 weeks by cesarean section without complications. 40 Although interesting, further research is needed to evaluate the safety and potential benefits of uterine transposition.

### Potential Treatment Modalities

The study conducted by Bath et al. found that steroid treatment positively influenced uterine volume in women with ovarian failure. Four participants (age range at TBI: 4–14 years) with reduced uterine volume, undetectable blood supply, and an absent endometrium are reported. After 3 months of physiological sex steroid replacement therapy, uterine blood supply and endometrial response were comparable to those in the control group. While uterine volume showed improvement, it remained significantly smaller than that of the controls, and the degree of improvement correlated with the age at which the participants received TBI. Three patients treated prepubertally showed a larger improvement than the one patient treated postpubertally. These findings might suggest that steroid replacement therapy may lead to restore certain uterine functions in this population. 7 However, it is not known whether standard regimens of estrogen replacement therapy are sufficient to facilitate uterine growth in adolescent women treated with radiotherapy in childhood. Even if the uterus is able to respond to exogenous sex steroid stimulation, and appropriate ARTs are available, a successful pregnancy outcome is by no means ensured. 10

There is preliminary evidence suggesting that the addition of pentoxifylline and tocopherol (vitamin E) to hormone therapy may have a therapeutic effect on uterine function following radiation exposure. 41 Pentoxifylline and tocopherol are hypothesized to offer anti-inflammatory and antioxidant properties that could help in repairing radiation-induced cellular damage. Some evidence suggests that pentoxifylline may improve endometrial proliferation. 42 However, while these findings are interesting, further research is needed to validate their efficacy and determine optimal treatment protocols, as well as to assess any long-term outcomes or side effects. It remains uncertain whether these preliminary findings will ultimately translate into effective clinical treatments.

## Uterine Monitoring and Pregnancy Management

For women who conceive after pelvic radiotherapy, close monitoring of the pregnancy is essential to identify and manage potential complications early. Regular ultrasounds can help assess uterine function, placental health, and fetal growth. High-risk pregnancy specialists may also recommend interventions to prevent preterm labor, such as cervical cerclage or the use of progesterone supplements to support the pregnancy. Cardiomyopathy surveillance is advised for all female survivors treated with anthracyclines and chest radiation, preferably preconceptionally or in the first trimester of the pregnancy. 20

In some cases, women with a history of pelvic radiotherapy may require delivery via cesarean section, particularly if placental abnormalities (such as placenta accreta spectrum) are present. This approach minimizes the risk of postpartum hemorrhage and other complications during vaginal delivery. It must be noted, however, that most women will be able to deliver vaginally in a safe matter. A gap in current knowledge remains regarding which patients are exactly at most risk, at what timing induction of labor would possibly be beneficial, and which extensive monitoring is beneficial and cost-effective.

## Conclusion

A well-known long-term effect of oncological treatment is POI, but another critical and sometimes overlooked organ at risk in female cancer survivors is the uterus. The impact of radiotherapy on the uterus is profound, particularly for women prior to or of reproductive age who wish to preserve their fertility and pursue pregnancy posttreatment. Radiotherapy can cause significant damage to the uterine structure and function, including fibrosis, vascular damage, and hormonal disruption. These changes increase the risk of infertility, miscarriage, low birth weight, and other pregnancy complications ( Fig. 1 ).

Advancements in fertility preservation techniques and ARTs have provided hope for women affected by pelvic radiotherapy. However, protecting or preserving oocytes is only one step; the next is protecting the uterus so that it can safely grow an embryo and fetus.

Further research is needed to develop more effective ways to protect the uterus from radiation damage and improve pregnancy outcomes for cancer survivors. With continued innovation in cancer treatment and fertility preservation, women can have a better chance of achieving successful pregnancies and maintaining their reproductive autonomy after cancer treatment. It is essential to ensure that pregnant individuals with a history of radiotherapy receive comprehensive care and support throughout their pregnancies. This includes not only regular monitoring of maternal and fetal health but also specialized assessments that take into account the potential effects of previous radiation exposure. Healthcare providers should be well-informed about the unique challenges faced by these individuals, enabling them to offer tailored guidance on managing risks associated with their medical history.
